# Relationship Between Social Support and Body Mass Index Among Overweight and Obese African American Women in the Rural Deep South, 2011–2013

**DOI:** 10.5888/pcd11.140340

**Published:** 2014-12-24

**Authors:** Erica R. Johnson, Tiffany L. Carson, Olivia Affuso, Claudia M. Hardy, Monica L. Baskin

**Affiliations:** Author Affiliations: Erica R. Johnson, Tiffany L. Carson, Olivia Affuso, Claudia M. Hardy, University of Alabama at Birmingham, Birmingham, Alabama.

## Abstract

**Introduction:**

African American women in the Deep South of the United States are disproportionately obese, a condition strongly influenced by their social environment. The objective of this study was to characterize the prevalence of social support from family and friends for healthy eating and exercise in rural communities.

**Methods:**

This study is an analysis of a subgroup (N = 195) of overweight and obese African American women from a larger ongoing weight loss trial (N = 409) in rural communities of the Alabama Black Belt and Mississippi Delta. The Social Support and Eating Habits Survey and Social Support and Exercise Survey were used to measure support from family and friends for healthy eating and exercise, respectively. Linear regression was conducted to determine the association between social support factors and body mass index (BMI).

**Results:**

Concurrently prevalent in our sample were encouraging support for healthy eating (family, median,14.0; range, 5.0–25.0; friends, median, 13.0; range 5.0–25.0) and discouraging support for healthy eating (family, median, 12.0; range, 5.0–25.0; friends, median, 11.0; range, 5.0–25.0). Median scores for support for exercise received in the form of participation from family and friends were 24.0 (range 10.0–48.0) and 24.0 (range 10.0–50.0), respectively. The median score for support for exercise in the form of rewards and punishment from family was 3.0 (range, 3.0–11.0). Social support factors were not associated with BMI.

**Conclusion:**

Overweight and obese African American women in the rural Deep South experience minimal social support from family and friends for healthy eating and exercise. Given the evidence that social support promotes healthy behaviors, additional research on ways to increase support from family and friends is warranted.

## Introduction

Obesity prevalence in the United States is higher in rural than in urban areas, particularly among people from racial/ethnic minorities. An estimated 40% of rural residents, compared with 33% of urban residents, are obese (body mass index [BMI] ≥30 kg/m^2^). Likewise, 56% of African American rural residents are obese compared with 43% of African American urban residents and 38% of white rural residents (1). Additional geographic disparities include greater obesity prevalence among residents of the South relative to other regions of the United States (1,2).

African American women residing in the Deep South states of Alabama, Georgia, Louisiana, Mississippi, and South Carolina are disproportionately burdened by obesity, physical inactivity, and poor-quality diets (2–5); therefore, there is an urgent need to more fully understand what influences the behaviors of this group at the individual, interpersonal, communal, and societal levels (6). Most weight loss interventions that target the modification of individual factors have been conducted mostly in white and urban populations; these studies demonstrated short-term weight reductions among African American women, although the reductions were less than those for women of other races as an effect of the intervention among African American women when compared with other racial groups (7).

Prior studies of various populations found no cross-sectional association between social support and BMI; however, this relationship has not been examined among African American women in the rural Deep South where the negative effects of certain social circumstances (eg, high rates of unemployment and poverty, low educational levels, limited access to health care, high risk for chronic disease) (8–11) may be lessened with appropriate social support. Social support for healthy eating and exercise, both elements of the social environment, are suggested as essential components of weight loss and weight management for residents in rural communities (12,13). Studies also suggest that people attempting weight loss rely on support from family and friends as a primary source of encouragement (8,14,15). Despite the potential benefits of social support for weight loss or weight management, few studies examined the prevalence of social support for healthy eating and exercise in a subgroup at great risk of obesity and other chronic conditions: African American women. The objective of this study was to characterize the social support environment for overweight and obese African American women residing in Deep South rural communities and to examine its association with BMI.

## Methods

### Setting

The target population consisted of people living in counties associated with an ongoing academic–community partnership to eliminate disparities in cancer rates between African Americans and whites in Alabama and Mississippi. The Deep South Network for Cancer Control operates in rural and urban areas that are densely populated by African Americans and uses a community-based participatory research approach to achieve its goal (16). For this study, all procedures were conducted in rural communities of the Alabama Black Belt and the Mississippi Delta, both of which are characterized by high prevalence of unemployment and poverty, poor access to health care, and low-density population (16).

### Participants and study design

This study is an analysis of a subgroup of participants from an ongoing study testing the efficacy of a culturally relevant weight loss program for African American women living in rural communities in the Deep South. Participants in the parent study (N = 409) identified as being African American were overweight or obese (BMI ≥25) at baseline and lived in 1 of the 4 rural counties of the Alabama Black Belt or 1 of the 4 rural counties in the Mississippi Delta associated with the Deep South Network for Cancer Control. Communities were randomized to receive either a weight loss intervention (2 in Alabama and 2 in Mississippi) or a weight loss intervention plus community strategies to support weight loss (2 in Alabama and 2 in Mississippi). Recruitment for the parent study was conducted from January 2011 through September 2013.

For the current study, only baseline information for people with complete data for demographic, clinical, and social support variables was used (n = 195). Sensitivity analysis revealed that missing data did not occur at random; therefore, complete case analysis was conducted, which showed that participants with complete data for all social support variables did not differ significantly in demographic characteristics from participants without complete data.

The data were analyzed to determine the prevalence of social support for healthy eating habits and exercise and to examine the association between BMI and social support for healthy eating habits and exercise. All recruitment procedures and study methods were approved by the institutional review board at the University of Alabama at Birmingham.

### Demographic measures

A survey was used to capture demographic information. Age was recorded in years. Employment status was categorized: employed versus unemployed. Annual household income was categorized as less than $10,000, $10,000 to $29,999, $30,000 to $49,999, and $50,000 or more. Highest level of education completed was categorized as less than high school, high school graduate or general education development certificate, or more than high school graduate. Marital status was categorized as married, no longer married, never married.

### Clinical measures

Participants’ weight was measured with a professional digital scale (SECA 2-in-1 Model #8761321004) to the nearest 0.1 kg while wearing light-weight clothing, and without shoes. Participants’ height was measured to the nearest 0.1 cm without shoes using a portable stadiometer (SECA 2-in-1 Model #8761321004). BMI was calculated from measured height and weight (BMI = weight in kg divided by height [m^2^]). For this study, participants were categorized into 1 of the following 2 BMI categories: overweight/class 1 obesity (BMI 25–34.99) or class 2/class 3 obesity (BMI ≥35). These cut points were selected on the basis of research that suggests that African American women do not begin to show increased risk for various adverse health outcomes until they reach high BMI levels (17).

### Environmental measures

The Social Support and Eating Habits Survey (18) (10 items) and the Social Support and Exercise Survey (18) (13 items) were used to measure participants’ perceived support from family and friends for healthy eating and exercise. Each measure used a 5-point Likert-type scale (1= none, 2 = rarely, 3 = a few times, 4 = often, 5 = very often) to indicate the frequency of social support provided by family and friends in the previous 3 months (18). Both surveys showed internal consistency (Cronbach’s α = 0.61–0.91) and test-retest reliability (*r* = 0.55–0.86) (18).

For the Social Support and Eating Habits Survey, composite scores representing encouraging (the sum of items 1–5) and discouraging (the sum of items 6–10) support for healthy eating were created and calculated separately for family and friends. Examples of survey items representing encouraging support for healthy eating included “Encouraged me not to eat unhealthy foods when I’m tempted to do so” and “Reminded me not to eat high fat, high salt foods,” while discouraging support for healthy eating included “Ate high fat or high salt foods in front of me” and “Offered me food I’m trying not to eat.”

For the Social Support and Exercise Survey, we created composite scores representing participation (the sum of items 11–16 and 20–23) and rewards and punishment (the sum of items 17–19) intended to be scored for family only (18) for exercise. Examples of survey items representing support in the form of participation in exercise included “Exercised with me” and “Helped plan activities around my exercise”; rewards for exercise included “Gave me rewards for exercising,” and punishment included “Criticized me or made fun of me for exercising.”

### Statistical analysis

Frequencies were reported for categorical variables: employment, income, education, and marital status. Descriptive statistics were reported for continuous variables: age, BMI, and social support composite scores. Wilcoxon rank sum nonparametric tests were used to evaluate differences in continuous demographic variables and social support scores by obesity status. Fisher’s exact tests were used to examine differences in categorical demographic variables by obesity status. Wilcoxon signed-rank nonparametric tests were used to examine differences in social support scores for support received from family compared with friends.

Although counties were selected at random for the intervention, no significant differences were found between counties as a result of the clustered study design; therefore, general linear regression was used to examine the association between each social support composite score and BMI, with separate models for family and friends. Multivariable linear regression analysis was conducted to control for county, education, employment status, income, and marital status. All analyses were conducted using SAS version 9.2 (SAS Institute, Inc). Significance was set at *P* < .05.

## Results

### Sample characteristics

The median age for the sample was 44.0 years (Table 1). Most participants attained a level of education beyond high school (62.6%), were not married (63.1%), earned less than $29,999 a year (63.6%), and were employed (72.8%). No significant differences in demographic characteristics were observed by obesity status. Descriptive statistics for social support composite scores for healthy eating and exercise are presented in Table 2 and are described in further detail below.

**Table 1:** Demographic and Clinical Characteristics of Study Population Stratified by Body Mass Index (N = 195), Deep South Network for Cancer Control(16), 2011–2013

Characteristic	All (N = 195)	BMI 25.0–34.99 (N = 70)	BMI ≥35 (N = 125)	*P* Value
**Age**				
Mean (SD)	45.0 (9.8)	46.3 (10.5)	44.3 (9.6)	.20^a^
Median (range)	44.0 (30.0–69.0)	48.0 (30.0–66.0)	43.0 (30.0–69.0)	
**Education, N (%)**				
<High school	8 (4.1)	3 (4.3)	5 (4.0)	.50
High school/GED	65 (33.3)	28 (40.0)	37 (29.6)	
>High school	122 (62.6)	39 (55.7)	83 (66.4)	
**Marital status, N (%)**				
Married	72 (36.9)	24 (34.3)	48 (38.4)	.94
No longer married	58 (29.8)	20 (28.6)	38 (30.4)	
Never married	65 (33.3)	26 (37.1)	39 (31.2)	
**Income, N (%)**				
<$10,000	39 (20.0)	18 (25.7)	21 (16.8)	.62
$10,000-$29,999	85 (43.6)	29 (41.4)	56 (44.8)	
$30,000-$49,999	48 (24.6)	15 (21.4)	33 (26.4)	
≥$50,000	23 (11.8)	8 (11.4)	15 (12.0)	
**Employment, N (%)**				
Employed	142 (72.8)	47 (67.1)	95 (76.0)	.23
Unemployed	53 (27.2)	23 (32.9)	30 (24.0)	

Abbreviation: BMI, body mass index; GED, general education development certificate; SD, standard deviation.

a For age, Wilcoxon rank sum nonparametric test was used to obtain *P* value, comparing participants with BMI 25.0–34.99 to those with BMI ≥35. All other *P* values were obtained by a Fisher’s exact test, comparing participants with BMI 25.0–34.99 to those with BMI ≥35.

**Table 2:** Effect of Support From Family Versus Friends for Healthy Eating Habits and Exercise, by Body Mass Index (N = 195), Deep South Network for Cancer Control (16), 2011–2013

Variable	All (N = 195)		BMI 25.0–34.99 (N = 70)		BMI >35 (N = 125)		*P* Value^a^
	Median (Range)	Mean (SD)	Median (Range)	Mean (SD)	Median (Range)	Mean (SD)	
**Support for healthy eating habits**							
Encouragement							
Family	14.0 (5.0–25.0)	13.8 (5.7)	14.0 (5.0–25.0)	13.7 (5.8)	14.0 (5.0–25.0)	13.9 (5.7)	.86
Friends	13.0 (5.0–25.0)	12.9 (5.6)	12.5 (5.0–25.0)	12.8 (5.7)	13.0 (5.0–25.0)	13.0 (5.6)	.84
*P* value^b^	.02	.18	.05	NA
Discouragement							
Family	12.0 (5.0–25.0)	12.8 (4.9)	13.0 (5.0–24.0)	13.2 (4.8)	12.0 (5.0–25.0)	12.6 (4.9)	.36
Friends	11.0 (5.0–25.0)	11.6 (4.7)	11.0 (5.0–25.0)	12.1 (4.8)	11.0 (5.0–23.0)	11.4 (4.6)	.32
*P* value^b^	<.001	.05	.001	NA
**Support for exercise**							
Participation							
Family	24.0 (10.0–48.0)	24.9 (10.6)	22.5 (10.0–46.0)	24.4 (11.2)	25.0 (10.0–48.0)	25.1 (10.3)	.62
Friends	24.0 (10.0–50.0)	24.2 (11.1)	26.0 (10.0–49.0)	26.1 (11.5)	22.0 (10.0–50.0)	23.2 (10.8)	.09
*P* value^b^	.23	.32	.03	NA
Reward or punishment^c^							
Family	3.0 (3.0–11.0)	4.3 (1.8)	3.0 (3.0–10.0)	4.3 (1.8)	3.0 (3.0–11.0)	4.3 (1.8)	.88

Abbreviations: BMI, body mass index; NA, not applicable; SD, standard deviation.

a Wilcoxon rank sum nonparametric test was used to obtain *P* value, comparing participants with BMI 25.0–34.99 with those with BMI ≥35.

b Wilcoxon signed-rank nonparametric test was used to obtain *P* value, comparing support received from family to support received from friends.

c We did not compare the family’s level of support for exercise (reward or punishment) with friends’ level of support (18).

### Encouragement for healthy eating habits

Encouraging support for healthy eating was as follows: family, median 14.0 (range, 5.0–25.0); friends, median 13.0 (range, 5.0–25.0) with no significant differences observed by obesity status (*P* = .86 for family and *P* = .84 for friends). Encouraging support for healthy eating received from family was significantly higher than from friends among participants with a BMI at or greater than 35 (*P* = .05). In the multivariable models, there was no significant association between BMI and encouraging support (family, *F*[16,178] = 0.72, *R* ^2^ = 0.06, *P* = .77; friends, *F*[16,178] = 0.72, *R* ^2^ = 0.06, *P* = .77).

### Discouragement for healthy eating habits

Discouragement for healthy eating was as follows: family, median 12.0 (range, 5.0–25.0); friends, median 11.0 (range, 5.0–25.0) with no significant differences observed by obesity status (*P* = .36 for family and *P* = .32 for friends). Discouragement for healthy eating from family was significantly higher than from friends for all participants (*P* < .001) and for both obesity categories (BMI = 25.0–34.99, *P* = .05; BMI ≥35, *P* = .001). In the multivariable models, there was no significant association between BMI and discouragement (family, *F*[16,178] = 0.74, *R* ^2^ = 0.06, *P* = .75; friends, *F*[16,178] = 0.77, *R* ^2^ = 0.06, *P* = 0.72).

### Participation in exercise

Support received in the form of participation in exercise was as follows: family, median 24.0 (range, 10.0–48.0; *P* = .62); friends, median 24.0 (range, 10.0–50.0, *P* = .09) with no significant differences observed by obesity status. However, support received from friends in the form of participation in exercise was greater among participants with a BMI from 25.0 to 34.99 than among those with a BMI at or greater than 35 for exercised with me (*P* = .04) and changed their schedule so we could exercise together (*P* = .01). Additionally, participants with a BMI at or greater than 35 received greater support in the form of participation in exercise from family than from friends (*P* = .03). However, in the multivariable models, there was no significant association between BMI and support received in the form of participation in exercise (family, *F*[16,178] = 0.71, *R* ^2^ = 0.06, *P* = .78; friends: *F*[16,178] = 0.97, *R* ^2^ = 0.08, *P* = .50).

### Rewards and punishment for exercise

The rewards and punishment received for exercise scale (median, 3.0; range, 3.0–11.0) was scored for family only (18). There were no significant differences between obesity categories (*P* = .88) for this construct.

## Discussion

Healthy eating and exercise are primary prescriptions for weight management and risk reduction for a myriad of chronic conditions. However, African American women and residents of rural communities are less likely than white women or urban residents to participate in these health-promoting behaviors (2,4,5). Unique barriers for African American rural residents, including limited community resources and long physical distance to resources, are documented (11,19,20). Furthermore, family and friends are an integral source of support for those attempting weight loss no matter where they live (14,15). As such, it is essential to understand the extent to which each of these sources of support (family, friends) influences obesity-prevention behaviors. This study is among the first to evaluate the prevalence of social support for obesity prevention behaviors among overweight and obese African American women living in the rural Deep South.

Our findings indicate that both family and friends concurrently encourage and discourage healthy eating. This finding is consistent with previous work that showed that positive and negative influences are independent of each other rather than inversely linked (18,21,22). For populations at high risk for being overweight or obese, such as people in Deep South rural communities, it is critical to minimize sources of discouragement for healthy eating. Sources of discouragement may arise from various circumstances that may include an effort to accommodate food preferences for other household family members while attempting to make healthier choices for oneself. In addition, discouragement may arise from the difficulty of selecting healthy foods at social gatherings, which are frequent in rural communities (23), with friends who do not have the same weight loss goals. Research shows that social support can lead to improved diet (24). A study of older rural women showed that the most frequently reported support for healthy eating received from both family and friends included positive feedback regarding changes in eating habits and encouragement for making healthy food choices when tempted with unhealthy choices (10). Thus, to help overweight people lose weight, it is important to provide an environment that encourages healthy eating. Women in this target population seeking to lose weight may benefit from socializing with peers who promote healthy eating, offer positive feedback for meeting goals, and generally cheer on their efforts toward behavior change. Although sources of support should ideally come from naturally occurring social networks, trained lay health advisors who are part of health promotion interventions are also effective in helping promote positive changes in diet behavior (25).

Our findings on social support for exercise in the form of participation by family and friends were similar to findings observed in a previous study that examined the relationship between social support and exercise among older adults (mean family social support, 21.8; mean friend social support, 24.1) (26). Moreover, when friends were asked to exercise with our study participants and also when friends changed their schedule so that they could exercise with study participants, results showed that friends provided greater support for participants in the overweight/class I obesity category than for those in the class II/class III obesity category. These findings are similar to those from a study of older rural women, which showed that the most frequently reported support for participation in exercise from both family and friends included offers to participate in exercise and encouragement to adhere to the exercise regimen (10). However, a study in which most participants were African American rural residents produced mixed results: both encouragement as well as the lack of encouragement for support in the form of family participation in exercise (14). Our findings that friends provided greater support for people at low obesity levels may be attributable to the perception of weight loss attainability; that is, friends may be more willing to participate in physical activity efforts when they perceive the weight loss goal to be more easily attainable. Or it may be that participants at high obesity levels are less receptive to receiving support from any source because of beliefs that weight loss goals can be reached independently (14). Kegler and colleagues noted that some participants lacked the desire to receive support for exercise (14). Although we found no linear relationship between social support and BMI, previous research has shown that friends’ support can be linked to increased exercise behaviors (26). Nevertheless, our results indicate a need for more efforts to increase support for exercise in rural communities for those attempting weight loss. Potential strategies include the development of physical activity groups among naturally occurring social networks (eg, friends, family, civic and social clubs, faith-based groups) or participation in health promotion interventions with group-based or partnered physical activity. For example, our prior work suggests a relationship between long-term participation in a physical activity intervention and improved health behaviors among rural residents who were connected to a community-based health promotion network (27).

Our results indicated that family members were more supportive than friends for obesity prevention behaviors. These findings are consistent with a study that reported more support received from family for both healthy eating and exercise in a sample of older rural women (10). Studies demonstrated the influential role of family members as a primary source of support for lifestyle changes for eating habits and physical activity (8,9,14). Our results underscore the family’s contribution to the social environment of overweight and obese African American rural women attempting weight loss, particularly those who are severely obese. Our results indicated that although friend support for healthy eating and exercise may be lesser than support received from family in this sample, both sources involve essential components of the social support environment for those attempting weight loss in rural communities. Thus, weight-loss interventions should focus on increasing support from both family and friends because these sources are the most likely to provide support for obesity prevention behaviors (28). Future studies should evaluate reasons for differences between support from family and support from friends in rural communities.

This study had limitations. First, the cross-sectional design limited the ability to provide causal inference. Second, no official scoring approach for the survey has been designated as the preferred method nor have any practically meaningful cutpoints been established, which limited our ability to compare results across studies or to provide qualitative descriptions of our study sample. Third, the study population comprised a unique population of overweight and obese African American women from a larger weight loss intervention, thus reducing the generalizability to other populations and restricting the BMI range. 

Our study had several strengths. It differentiated between specific components of family and friend social support for healthy eating and exercise, and it used a unique, underrepresented population, which may lead to the creation of interventions targeted specifically for this population. A complete case analysis reduced the maximum sample size; however, our sample size was still large in comparison with samples in studies of other weight-loss interventions. Finally, the use of measured height and weight for calculating BMI rather than reliance on self-report increased the accuracy of this measure.

It is important to identify and foster positive support for healthy eating and exercise among overweight and obese African American women in rural communities in the Deep South. People with training on how to elicit social support from support partners who have also received training on how to provide social support related to healthier eating habits and exercise achieve greater weight losses than do people attempting to lose weight alone (29). However, because much research has been conducted in white and urban populations, it remains unclear how social support from family members and friends affects eating behaviors, exercise, and weight loss in African American women living in rural communities. Our results indicate that more effort should be invested in providing social support networks for those attempting weight loss in rural communities. Future research should determine longitudinal associations between social support and weight loss in rural communities of the Deep South.
